# Genomewide Association Study of Acute Anterior Uveitis Identifies New Susceptibility Loci

**DOI:** 10.1167/iovs.61.6.3

**Published:** 2020-06-03

**Authors:** Xiu-Feng Huang, Zhixiu Li, Erika De Guzman, Philip Robinson, Lianne Gensler, Michael M. Ward, Mohammad Hossein Rahbar, MinJae Lee, Michael H. Weisman, Gary J. Macfarlane, Gareth T. Jones, Eva Klingberg, Helena Forsblad-d'Elia, Peter McCluskey, Denis Wakefield, Jeff S. Coombes, Maria A. Fiatarone Singh, Yorgi Mavros, Nicole Vlahovich, David C. Hughes, Helena Marzo-Ortega, Irene Van der Horste-Bruinsma, Finbar O'Shea, Tammy M. Martin, James Rosenbaum, Maxime Breban, Zi-Bing Jin, Paul Leo, John D. Reveille, B. Paul Wordsworth, Matthew A. Brown

**Affiliations:** 1 Translational Genomics Group, Institute of Health and Biomedical Innovation, Queensland University of Technology at Translational Research Institute, Princess Alexandra Hospital, Brisbane, Australia; 2 School of Optometry and Ophthalmology and Eye Hospital, Wenzhou Medical University, Wenzhou, Zhejiang, China; 3 State Key Laboratory of Optometry, Ophthalmology and Vision Science, Wenzhou, Zhejiang, China; 4 University of Queensland School of Clinical Medicine, Royal Brisbane Hospital, Herston Queensland, Australia; 5 Department of Medicine/Rheumatology, University of California San Francisco, San Francisco, California, United States; 6 Intramural Research Program, National Institute of Arthritis and Musculoskeletal and Skin Diseases, National Institutes of Health, Bethesda, Maryland, United States; 7 Division of Clinical and Translational Sciences, Department of Internal Medicine, The University of Texas Health Science Center at Houston, Houston, Texas, United States; 8 David Geffen School of Medicine, University of California Los Angeles, Los Angeles, California, United States; 9 Epidemiology Group, Institute of Applied Health Sciences, School of Medicine, Medical Sciences and Nutrition, University of Aberdeen, Foresterhill, Aberdeen, Scotland, United Kingdom; 10 Aberdeen Centre for Arthritis and Musculoskeletal Health, University of Aberdeen, Foresterhill, Aberdeen, Scotland, United Kingdom; 11 Department of Public Health and Clinical Medicine, Rheumatology, Umeå University, Umeå, Sweden; 12 Department of Rheumatology and Inflammation Research, Sahlgrenska Academy at University of Gothenburg, Gothenburg, Sweden; 13 Discipline of Clinical Ophthalmology and Eye Health Faculty of Medicine and Health, The University of Sydney, Sydney, Australia; 14 School of Medical Sciences, University of New South Wales, Kensington, New South Wales, Australia; 15 School of Human Movement and Nutrition Sciences, University of Queensland, Brisbane, Australia; 16 Physical Activity, Lifestyle, Ageing and Wellbeing Faculty Research Group, Faculty of Health Sciences, University of Sydney, Sydney, New South Wales, Australia; 17 Department of Sports Medicine, Australian Institute of Sport, Bruce, ACT, Australia; 18 NIHR Leeds Biomedical Research Centre, Leeds Teaching Hospitals NHS Trust, Leeds, United Kingdom; 19 Leeds Institute of Rheumatic and Musculoskeletal Medicine, University of Leeds, Leeds, United Kingdom; 20 Department of Rheumatology, VU University Medical Centre, Amsterdam, The Netherlands; 21 Department of Rheumatology, St. James's Hospital, Dublin, Ireland; 22 Departments of Ophthalmology, Medicine, and Cell Biology, Oregon Health & Sciences University and Chair Emeritus, Legacy Devers Eye Institute, both in Portland, Oregon, United States; 23 Departments of Ophthalmology, and Molecular Microbiology & Immunology, Oregon Health & Sciences University, Oregon, United States; 24 UMR 1173, Inserm, University of Versailles Saint-Quentin, Montigny-le-Bretonneux, France; 25 Hôpital Ambroise Paré, AP-HP, and Université de Versailles Saint-Quentin, Boulogne-Billancourt, France; 26 INFLAMEX, Laboratoire d'Excellence, Université Paris Diderot, Sorbonne Paris Cité, France; 27 Division of Ophthalmic Genetics, The Eye Hospital, Wenzhou Medical University, Wenzhou, China; 28 State Key Laboratory of Ophthalmology, Optometry and Vision Science, National International Joint Research Center for Regenerative Medicine and Neurogenetics, Wenzhou Medical University, Wenzhou, China; 29 Rheumatology and Clinical Immunogenetics, University of Texas Health Science Center at Houston, Houston, Texas, United States; 30 NIHR Oxford Musculoskeletal Biomedical Research Unit, Nuffield Orthopaedic Centre, Headington, Oxford, United Kingdom; 31 Centre for Precision Medicine, The First Affiliated Hospital of Wenzhou Medical University, Wenzhou, China; 32 Guy's & St Thomas’ NHS Foundation Trust and King's College London NIHR Biomedical Research Centre, London, United Kingdom

**Keywords:** acute anterior uveitis, ankylosing spondylitis, GWAS, heritability, genetic risk scores

## Abstract

**Purpose:**

Acute anterior uveitis (AAU) is a common intraocular inflammatory disease. AAU occurs in 30% to 50% of patients with ankylosing spondylitis (AS), and both conditions are strongly associated with human leukocyte antigen (*HLA*)*-B27*, implying a shared etiology. This study aims to apply genomewide association study (GWAS) to characterize the genetic associations of AAU and their relationship to the genetics of AS.

**Methods:**

We undertook the GWAS analyses in 2752 patients with AS with AAU (cases) and 3836 patients with AS without AAU (controls). There were 7,436,415 single-nucleotide polymorphisms (SNPs) available after SNP microarray genotyping, imputation, and quality-control filtering.

**Results:**

We identified one locus associated with AAU at genomewide significance: rs9378248 (*P* = 2.69 × 10^−8^, odds ratio [OR] = 0.78), lying close to *HLA-B*. Suggestive association was observed at 11 additional loci, including previously reported AS loci *ERAP1* (rs27529, *P* = 2.19 × 10^−7^, OR = 1.22) and *NOS2* (rs2274894, *P* = 8.22 × 10^−7^, OR = 0.83). Multiple novel suggestive associations were also identified, including *MERTK* (rs10171979, *P* = 2.56 × 10^−6^, OR = 1.20), *KIFAP3* (rs508063, *P* = 5.64 × 10^−7^, OR = 1.20), *CLCN7* (rs67412457, *P* = 1.33 × 10^−6^, OR = 1.25), *ACAA2* (rs9947182, *P* = 9.70 × 10^−7^, OR = 1.37), and 5 intergenic loci. The SNP-based heritability is approximately 0.5 for AS alone, and is much higher (approximately 0.7) for AS with AAU. Consistent with the high heritability, a genomewide polygenic risk score shows strong power in identifying individuals at high risk of either AS with AAU or AS alone.

**Conclusions:**

We report here the first GWAS for AAU and identify new susceptibility loci. Our findings confirm the strong overlap in etiopathogenesis of AAU with AS, and also provide new insights into the genetic basis of AAU.

Uveitis is a major cause of ocular disease, leading to 5% to 10% of visual impairment worldwide.^1^ The prevalence varies depending on the ethnicity and geographic locations: 115 per 100,000 in America,^2^ 40 per 100,000 in Japan,^3^ and 310 to 730 per 100,000 in Southern India.^4^ Acute anterior uveitis (AAU) is the most common type of uveitis and is characterized by inflammation of the anterior chamber.^5^ The typical clinical manifestations of AAU are that of abrupt onset of unilateral, often alternating, anterior uveitis, with significant cellular and protein extravasation into the anterior chamber and tendency for recurrences.^6^ AAU is frequently associated with spondyloarthropathies, such as ankylosing spondylitis (AS), psoriatic arthritis, and inflammatory bowel disease.^7^ Among those spondyloarthropathies, AS is the most common associated condition, present in 30% to 50% of patients with AAU. AAU is strongly associated with human leukocyte antigen (HLA)-B27.^8^ A large genetic study reported the prevalence of HLA-B27 was 81.8% in the group with ophthalmologist-diagnosed AAU and 92.0% in the group with self-reported AAU.^9^ Among patients with AAU who are HLA-B27 positive, the prevalence of concomitant AS rises to 80% to 84%.^10^

Multiple studies have demonstrated that genetic components play a major role in uveitis. Derhaag et al. found that the prevalence of AAU in HLA-B27-positive first-degree relatives of patients with AAU was 13%, significantly higher than the frequency of 1% in the HLA-B27-positive individuals without affected relatives, indicating high familiality.^11^ Several polymorphisms have been identified to be associated with the presence of AAU. To date, the strongest genetic association between a genetic component and AAU is attributable to *HLA-B27*, the major histocompatibility complex (MHC) type I allele. On the basis of genotyping using the Illumina Immunochip, a comparison between AAU and healthy control subjects found significant association over *HLA-B*, corresponding to the *HLA-B27* tag single-nucleotide polymorphism (SNP) rs116488202.^9^ This study also found the association of three non-MHC loci, *ERAP1*, *IL23R*, and the intergenic region 2p15 with genomewide significance, and five loci reached a suggestive level of significance (*IL10-IL19*, *IL18R1-IL1R1*, *IL6R*, the chromosome 1q32 locus harboring *KIF21B*, and a retinal-related gene *EYS*).^9^ The authors also demonstrated significant differences in effect size of several previously discovered AS loci between AS+AAU+ and AS+AAU-, using two different models. In the first model (including the SNP and principal components), different effect sizes were observed in *ERAP1*, *UBE2LE*, *ICOSLG*, and *EYS*. In the second model (including the SNP, principal components, and *HLA-B27* dose), different effect sizes were observed in *ERAP1*, *ANTXR2*, 21q22, and 2p15.^9^ Additionally, in an analysis comparing patients with AS with AAU versus controls, *HLA-B27* and HLA-A*0201 were strongly associated with AAU using Illumina Exomechip microarray.^12^ Many candidate-gene association studies have reported nominal SNP associations in interleukin (IL) genes, tumor necrosis factor (TNF) genes, and complement factors correlated with anterior uveitis.^13^^–^^15^ However, those candidate-gene association studies were not adequately powered to reliably identify genes involved in AAU, nor did they control for potential population stratification effects, and, thus, their interpretation is unclear.

Genomewide association studies (GWAS) have proven to be a powerful and robust method to identify genetic associations with complex diseases over the past decade. For example, using GWAS, more than 100 AS susceptibility loci have been identified,^16^^–^^20^ which have been transformational in understanding the pathogenesis of AS,^21^^,^^22^ have changed disease management through repositioning of IL-17 inhibitors for management of the disease,^23^ and have stimulated several drug development programs.^24^ Although GWAS have been undertaken for nearly all major immune-mediated diseases, at this point, there have been no GWAS for AAU. This study aims to apply GWAS to characterize extensively the genetic associations of AAU and their relationship to the genetics of AS.

## Methods

### Sample Collection and Genotyping

AS case and control cohorts involved in this study are described in Supplementary Table S1. AS cases were defined by the modified New York criteria.^25^ Control participants were not specifically screened either for AS or AAU. All patients gave written informed consent, and ethics approval has been obtained from all relevant institutional ethics committees.

### Genotyping and Quality Control

All the individuals of European descent have been previously genotyped using Illumina Infinium HumanCoreExome 24v1.1, according to the manufacturer's recommendations. Subsequently, bead intensity data were processed and normalized for each sample in Illumina GenomeStudio software. Data for successfully genotyped samples were extracted, and genotypes were called within collections using GenomeStudio. The human genome build 19 was used (UCSC).

The thresholds used in quality control include: a genotyping missingness rate of 0.05; an individual missingness rate of 0.05; a Hardy-Weinberg Equilibrium (HWE) threshold in controls of 1 × 10^−6^; a minor allele frequency (MAF) of 0.01; heterozygosity versus missingness outliers beyond three SDs were excluded; identity by descent (IBD) threshold of PI-HAT (proportion [IBD = 2] + 0.5 [IBD = 1]) 0.185 was used. Principal components analysis (PCA) was then computed using Genome-wide Complex Trait Analysis (GCTA) after the removal of regions of long-range linkage disequilibrium.^26^ Ancestry outliers were removed by using the top two components. A second round of PCA was then performed to better resolve ancestry differences within the cohorts. Principal components were used as covariates to control for population stratification.

### Imputation

Genotyping data were imputed using Sanger imputation service (https://imputation.sanger.ac.uk/). Optional pre-phasing was with EAGLE2^27^ and imputation with PBWT.^28^ The Haplotype Reference Consortium was used as the references panel. Imputed loci with quality score < 0.6 were excluded from the association testing. Detailed investigations of the MHC alleles and HLA loci were performed using SNP2HLA,^29^ which is an analysis package that performs HLA allele and amino acid imputation from SNP data and association analysis.

### Association Analysis

Plink (https://www.cog-genomics.org/plink2)^30^ was used to perform association analyses with eigenvectors from PCA as covariates for population stratification control. Significance levels were defined as genomewide (*P* < 5 × 10^−8^) or suggestive when *P* > 5 × 10^−8^ but < 1 × 10^−5^. Previous reported loci were also examined. Subsequently, conditional analysis for secondary signal detection was performed in all susceptibility loci by fitting the primary SNP as a fixed effect. Manhattan plots and quantile to quantile (Q-Q) plots were displayed, and genomic inflation factor 1000 values were calculated. To identify the genetic basis of AAU, we designed the GWAS based on comparison of patients with AS with AAU (AS+AAU+) versus patients with AS without AAU (AS+AAU-). Locus zoom plotting was carried out using LocusZoom.^31^

### Estimation of SNP-Based Heritability

We estimated the SNP-based heritability (SNP *h*^2^) by GCTA.^26^ Heritability is the proportion of the phenotypic variance accounted for by genetic effects. We investigated SNP-based heritability by designing three different comparisons: (1) patients with AS with AAU (AS+AAU+) versus patients with AS without AAU (AS+AAU-); (2) patients with AS without AAU (AS+AAU-) versus controls; and (3) patients with AS with AAU (AS+AAU+) versus controls. The estimation of heritability was performed for a range of disease prevalences.

### Mendelian Randomization Analysis

To determine the most likely causal genes, we performed the summary data-based Mendelian randomization (SMR^32^) analysis for AAU with expression quantitative trait loci (eQTL) data. GWAS data consisted of patients with AS with AAU (AS+AAU+) and patients with AS without AAU (AS+AAU-), and the eQTL data was from the Consortium for the Architecture of Gene Expression (CAGE), which comprises individual-level whole-blood expression and genotype data on 2765 individuals.^33^ In the SMR analysis, only probes for which the *P* value of the top associated *cis*-eQTL was < 5 × 10^−8^ were included and the MHC region was excluded. To control the genomewide type I error rate, Bonferroni correction was used to account for multiple testing. Locus zoom plots of candidate loci were generated.

### Genomewide Polygenic Risk Scores

Polygenic risk scores (PRS) were calculated for each individual using the adaptive MultiBLUP algorithm.^34^ Only genotyped SNPs in common between all SNP arrays where the missingness rate was <0.05, the MAF was >0.01, and the HWE *P* value was > 1 × 10^−6^ were used. A conservative approach was used whereby the cohort was divided into independent training and test sets, rather than using a cross-validation approach.^35^ The training set was then used to calculate the scoring matrix. This MultiBLUP algorithm first selected regions based on a *P* values’ threshold (option-sig1) obtained using the training cohort. Within these regions, all SNPs with a significance threshold greater than a second *P* value threshold (option-sig2) were considered by the algorithm, which then controls for the linkage disequilibrium structure. The *P* value thresholds were optimized by choosing a range of values between 1 × 10^−7^ and 1 × 10^−3^ for option-sig1 and 1 × 10^−3^ and 1 × 10^−2^ for option-sig2. Then the resulting weighted predictors were applied to the test cohort to obtain per sample scores from which the maximum area under the curve (AUC) was obtained.

## Results

### Comparison of Patients With AS With AAU versus AS Alone

To investigate the genetic basis of AAU while controlling for concomitant AS, we performed a GWAS comparing patients with AS with AAU (AS+AAU+) versus patients with AS without AAU (AS+AAU-). After individual quality control (QC) there were 2752 patients with AS and with AAU and 3836 patients with AS alone, respectively. Given our sample size, the study has > 80% power for risk alleles of MAF = 0.2, for heterozygote odds ratios (ORs) > 1.21 at a type 1 error rate of 5 × 10^−8^. After imputation and SNP QC, there were 7,436,415 SNPs (Supplementary Table S2). Subsequently, analysis of logistic regression with principal components as covariates against linear mixed models was conducted. The top four eigenvectors were controlled because additional eigenvectors did not reduce the genomic inflation factor. Quantile to quantile (Q-Q) plots are presented in Supplementary Figure S1. Genomic inflation factor (λ) calculated using 7,396,528 SNPs (MHC SNPs were excluded) was 1.008 (λ_1000_ for an equivalent study of 1000 cases and 1000 controls = 1.002), indicating minimal evidence of residual population stratification in the overall data set. Genomic inflation factors stratified by frequency, imputation quality score, and MHC are shown in Supplementary Table S3.

**Figure 1.**
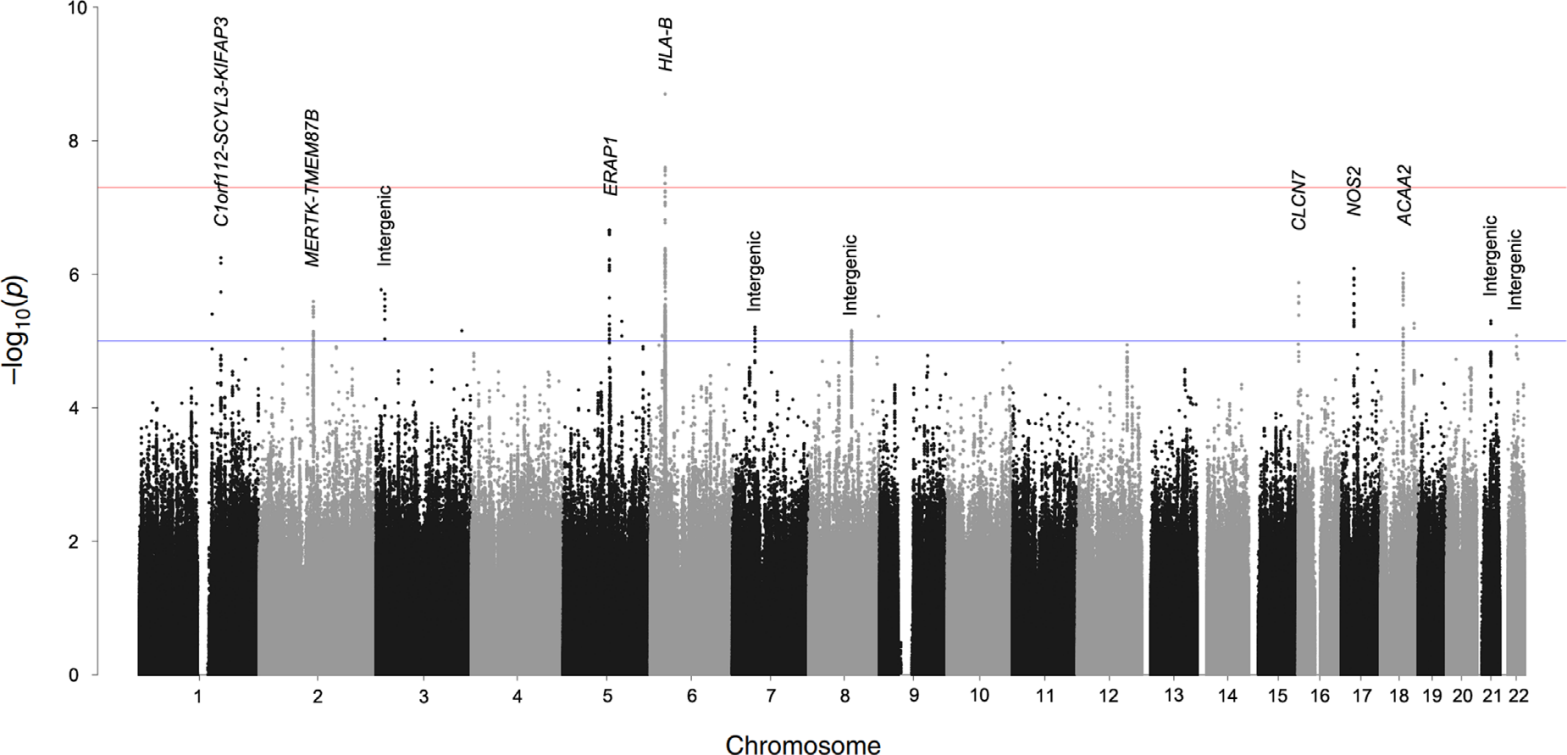
Manhattan plot of association analyses of patients with AS with AAU versus patients with AS without AAU. Y axis represents the *P* values on the -log10 scale. The red line represents *P* = 5 × 10^−8^ (genomewide significance), and the blue dashed line represents *P* = 1 × 10^−5^ (suggestive association).

**Figure 2.**
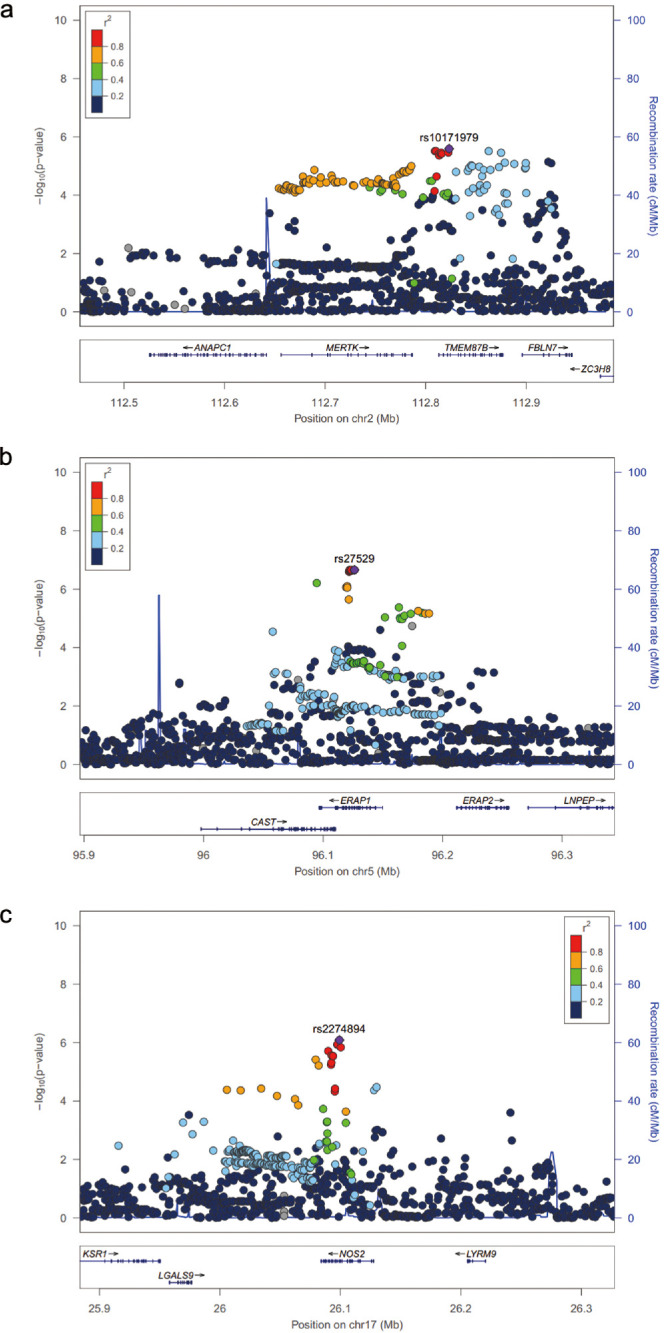
Locus zoom plot of association results for *MERTK-TMEM87B* locus (**a**), *ERAP1* locus (**b**), and *NOS2* locus (**c**). The reference population for LD data is 1000 Genomes EUR. SNPs with missing LD information are shown in grey.

We identified one locus associated with AAU at genomewide significance: rs9378248 (*P* = 2.69 × 10^−8^, OR = 0.78), lying close to *HLA-B*, a known susceptibility gene for both AAU and AS (Table 1 and Fig. 1). Suggestive association was observed at 11 additional loci summarized in Table 1. We found suggestive association with SNPs in *MERTK* locus (rs10171979, *P* = 2.56 × 10^−6^, OR = 1.20), which is also a novel finding in our ongoing GWAS in AS (Table 1 and Fig. 2a). Association was also observed with SNPs at previously reported AS loci, including *ERAP1* (rs27529, *P* = 2.19 × 10^−7^, OR = 1.22; Fig. 2b), and *NOS2* (rs2274894, *P* = 8.22 × 10^−7^, OR = 0.83; Fig. 2c).

**Table 1.** Results of the Association Analyses of Patients With AS with AAU versus patients with AS Without AAU

SNP ID	Chr.	Position^*^	Nearby Genes	*P* Value	Effect Allele	OR	95% CI	Associated With AS
rs9378248	6p21	31326289	*HLA-B*	2.69 × 10^−8^	A	0.78	0.71-0.85	Yes
rs508063	1q24	169923676	*C1orf112-SCYL3-KIFAP3*	5.64 × 10^−7^	A	1.20	1.12-1.28	No
rs10171979	2q13	112823320	*MERTK-TMEM87B*	2.56 × 10^−6^	C	1.20	1.11-1.30	Yes
rs76412624	3p24	18186605	Intergenic	1.98 × 10^−6^	G	0.67	0.57-0.79	No
rs27529	5q15	96126308	*ERAP1*	2.19 × 10^−7^	A	1.22	1.13-1.31	Yes
rs7784778	7p12	46951833	Intergenic	6.22 × 10^−6^	T	0.84	0.78-0.91	No
rs10093384	8q21	88635942	Intergenic	7.01 × 10^−6^	A	1.20	1.11-1.31	No
rs67412457	16p13	1525710	*CLCN7*	1.33 × 10^−6^	A	1.25	1.14-1.36	No
rs2274894	17q11	26099171	*NOS2*	8.22 × 10^−7^	T	0.83	0.78-0.90	Yes
rs9947182	18q21	47253904	*ACAA2*	9.70 × 10^−7^	T	1.37	1.21-1.55	No
rs7281081	21q21	29471489	Intergenic	4.98 × 10^−6^	T	0.79	0.71-0.87	No
rs1580226	22q12	34809453	Intergenic	8.26 × 10^−6^	T	0.82	0.75-0.89	No

Chr., chromosome; OR, odds ratio; 95% CI, 95% confidence interval.

* UCSC, human genome build 19.

In addition to these discoveries, association was seen at loci not previously known to be associated with AAU or AS, including *KIFAP3* (rs508063, *P* = 5.64 × 10^−7^, OR = 1.20), *CLCN7* (rs67412457, *P* = 1.33 × 10^−6^, OR = 1.25), *ACAA2* (rs9947182, *P* = 9.70 × 10^−7^, OR = 1.37), and five intergenic loci at 3p24 (rs76412624, *P* = 1.98 × 10^−6^, OR = 0.67), 7p12 (rs7784778, *P* = 6.22 × 10^−6^, OR = 0.84), 8q21 (rs10093384, *P* = 7.01 × 10^−6^, OR = 1.20), 21q21 (rs7281081, *P* = 4.98 × 10^−6^, OR = 0.79), and 22q12 (rs1580226, *P* = 8.26 × 10^−6^, OR = 0.82). Subsequently, conditional analysis for secondary signal detection was performed in all the 11 non-MHC loci by fitting the primary SNP as a fixed effect. After conditioning on these SNPs, no residual association was seen in these loci (*P* < 1 × 10^−4^), indicating no evidence in the current study for any secondary signal at these loci.

### Investigation of Reported AS Genes in AAU GWAS

To investigate further potential overlaps and differences between genetic associations of AAU and AS, we examined the associations between the causal genes of AS and AAU. AS genetic associations that either achieved genomewide significance in individual studies or as part of a cross-disease study of pleiotropic genes were included.^24^ If the reference SNP had been genotyped or imputed in the current study, the OR was compared directly. Where this was not the case, tagSNPs were identified, linkage disequilibrium with the reference SNP determined using LDlink (ldlink.nci.nih.gov), then ODs were compared taking into account the directionality of the linkage disequilibrium. In addition to *HLA-B*, *ERAP1*, *NOS2*, and *MERTK* (Table 1), moderate association was observed with SNPs in additional known AS genes at *IL23R*, *ASAP2*, *CMC1*, *IL12B*, *ZC3H12C*, *IL10*, *SP140*, and *PTPN2* (Supplementary Table S4). Furthermore, most of them showed the consistent direction of effect with the discoveries in AS GWAS (Supplementary Table S4, Supplementary Fig. S2). For example, rs11209032 in *IL23R* reported in AS GWAS with OR = 1.20,^18^ and this study showed OR = 1.14, with concordant direction of effect. In the *ASAP2* locus, rs2666218 was observed in AS with OR = 1.12, and was associated with AAU with OR = 1.13. Moreover, the top SNP in our AAU analyses, rs56111045, was in very strong linkage disequilibrium with it (D’ = 1; R^2^ = 0.995). These results indicate a consistent direction of effect of *ASAP2* locus in AS and AAU.

### HLA Imputation and Association Analysis

To better understand the genetic basis of the MHC susceptibility loci, we performed *HLA* imputation using SNP2HLA.^29^ After removing subjects with poor *HLA* imputation quality, 2725 patients with AS with AAU (AS+AAU+) and 3796 patients with AS without AAU (AS+AAU-) remained and were included in the analysis of MHC susceptibility. In total, 424 *HLA* alleles (including alleles at either two-digit or four-digit resolution) and 1276 amino acid residues were imputed. For the *HLA* alleles, as expected, HLA-B*27 (*P* = 1.86 × 10^−42^, OR = 2.35) was the most significantly associated allele with AAU (Table 2). The question of whether HLA-B*27 exerts its influence through a dominant or additive genetic model was assessed. Results showed that homozygosity for HLA-B*27 confers OR of 3.3 (95% confidence interval [CI] = 2.45–4.45), and heterozygosity for HLA-B*27 confers OR of 2.93 (95% CI = 2.53–3.4). This finding is consistent with previous studies of AS GWAS,^36^^,^^37^ suggesting that HLA-B*27 homozygosity increases risk over heterozygosity. In addition to HLA-B*27 alleles, genomewide significant association was also observed in unconditioned analyses with HLA-C*02 allele (Table 2), which was also reported in AS genetics.^38^ Nominal associations were also seen between AAU and previously AS-associated allele HLA-DRB1*0103,^38^ which is also known to be associated with inflammatory bowel disease.^39^^,^^40^ To detect whether other HLA alleles affect AAU susceptibility independently from the HLA-B*27, additional conditional analyses were performed. After adjusting for the HLA-B*27 allele, the next most-associated HLA allele was HLA-DRB1*15 (*P* = 8.14 × 10^−5^, OR = 1.28). After conditioning on both HLA-B*27 and HLA-DRB1*15, lead association was seen with HLA-DPB1*03 (*P* = 4.83 × 10^−3^, OR = 1.17).

**Table 2.** Association Analysis of HLA Alleles in the Comparison of Patients with AS with AAU versus Patients with AS without AAU

HLA Allele^#^	Position^*^	OR	95% CI	*P* Value
HLA-B*27	31431272	2.35	2.08-2.66	1.86 × 10^−42^
HLA-B*2705	31431272	2.27	2.02-2.56	1.49 × 10^−41^
HLA-C*02	31346171	1.33	1.21-1.45	3.37 × 10^−9^
HLA-C*0202	31346171	1.33	1.21-1.45	3.37 × 10^−9^
HLA-C*01	31346171	1.25	1.14-1.38	4.26 × 10^−6^
HLA-C*0102	31346171	1.25	1.14-1.38	4.26 × 10^−6^
HLA-DRB1*0103	32660042	1.34	1.14-1.57	0.00044
HLA-DRB1*1501	32660042	1.23	1.09-1.39	0.00074
HLA-C*0701	31346171	0.82	0.72-0.92	0.00078
HLA-B*4402	31431272	0.79	0.68-0.91	0.0011
HLA-C*05	31346171	0.80	0.70-0.91	0.0011
HLA-C*0501	31346171	0.80	0.70-0.91	0.0011
HLA-DQB1*0602	32739039	1.22	1.08-1.38	0.0018
HLA-DRB1*15	32660042	1.21	1.07-1.37	0.0018
HLA-B*08	31431272	0.81	0.71-0.93	0.0023
HLA-B*0801	31431272	0.81	0.71-0.93	0.0023
HLA-B*44	31431272	0.84	0.75-0.95	0.0051
Adjusting for the HLA-B*27 allele				
HLA-DRB1*15	32660042	1.28	1.13-1.45	8.14 × 10^−5^
HLA-B*07	31431272	1.30	1.13-1.50	0.00023
HLA-DPB1*03	33157346	1.16	1.04-1.29	0.0088
Adjusting for both HLA-B*27 and HLA-DRB1*15 allele				
HLA-DPB1*03	33157346	1.17	1.05-1.31	0.0048

OR, odds ratio; 95% CI, 95% confidence interval.

* UCSC, human genome build 19.

# HLA allele is imputed using SNP2HLA.

Considering amino acid residues, the most significant association was observed for amino acid position 97 in HLA-B (*P* = 6.09 × 10^−43^, OR = 2.36), which was also the strongest signal seen in AS study (Supplementary Table S5).^38^ Strong associations were also observed with the amino acid positions 70, 114, 77, 69, and 67 of HLA-B but all these signals lost significance (*P* > 1 × 10^−5^) conditioning on amino acid position 97 (Supplementary Table S5, Supplementary Table S6). After adjusting for the amino acid position 97, the most strongly associated signals were amino acid position 67 and 70 in HLA-B (*P* = 3.80 × 10^−5^, OR = 1.31; *P* = 4.42 × 10^−5^, OR = 1.31) and amino acid position 71 in HLA-DRB1 (*P* = 8.51 × 10^−5^, OR = 1.28; Supplementary Table S6).

### Interactions Between *ERAP1* and *HLA-B27*

Previous studies have observed that the association with the nonsynonymous SNP rs30187 (p.Lys528Arg) in the *ERAP1* locus is restricted to HLA-B*27-positive or HLA-B*40-positive *HLA-B27*-negative patients with AS.^36^^,^^38^ To assess the gene-gene interactions between *ERAP1* and *HLA-B27* in AAU, we investigated the associations by conducting two different comparisons. In the comparison between HLA-B*27-positive AS+AAU+ subjects and all AS+AAU- subjects, we observed significant association with rs30187 in *ERAP1* (*P* = 1.4 × 10^−8^, OR = 1.25). However, when we compared the HLA-B*27-negative AS+AAU+ subjects with all AS+AAU- subjects (*n* = 3796) or patients with HLA-B27-negative AS+AAU- (*n* = 927), we saw no association with rs30187 (*P* > 0.05). These observations further support the existence of an interaction between *ERAP1* and *HLA-B27*. The study did not have sufficient power to test the interaction between *HLA-B40* and *ERAP1* variants so this was not performed.

### eQTL Mendelian Randomization Analysis

To determine the most likely causal genes at associated loci, we performed the SMR^32^ analysis for AAU with eQTL data. GWAS data consisted of 2752 patients with AS with AAU (AS+AAU+) and 3836 patients with AS without AAU (AS+AAU-), and the eQTL data was from the CAGE, which comprises individual-level whole-blood expression and genotype data on 2765 individuals.^33^ In the SMR analysis, only probes for which the *P* value of the top associated *cis*-eQTL was < 5 × 10^−8^ were included and the MHC region was excluded. To control the genomewide type I error rate, Bonferroni correction was used to account for multiple testing, which resulted in a genomewide significance level of *P* = 5.95 × 10^−6^ (= 0.05/8403).

**Figure 3.**
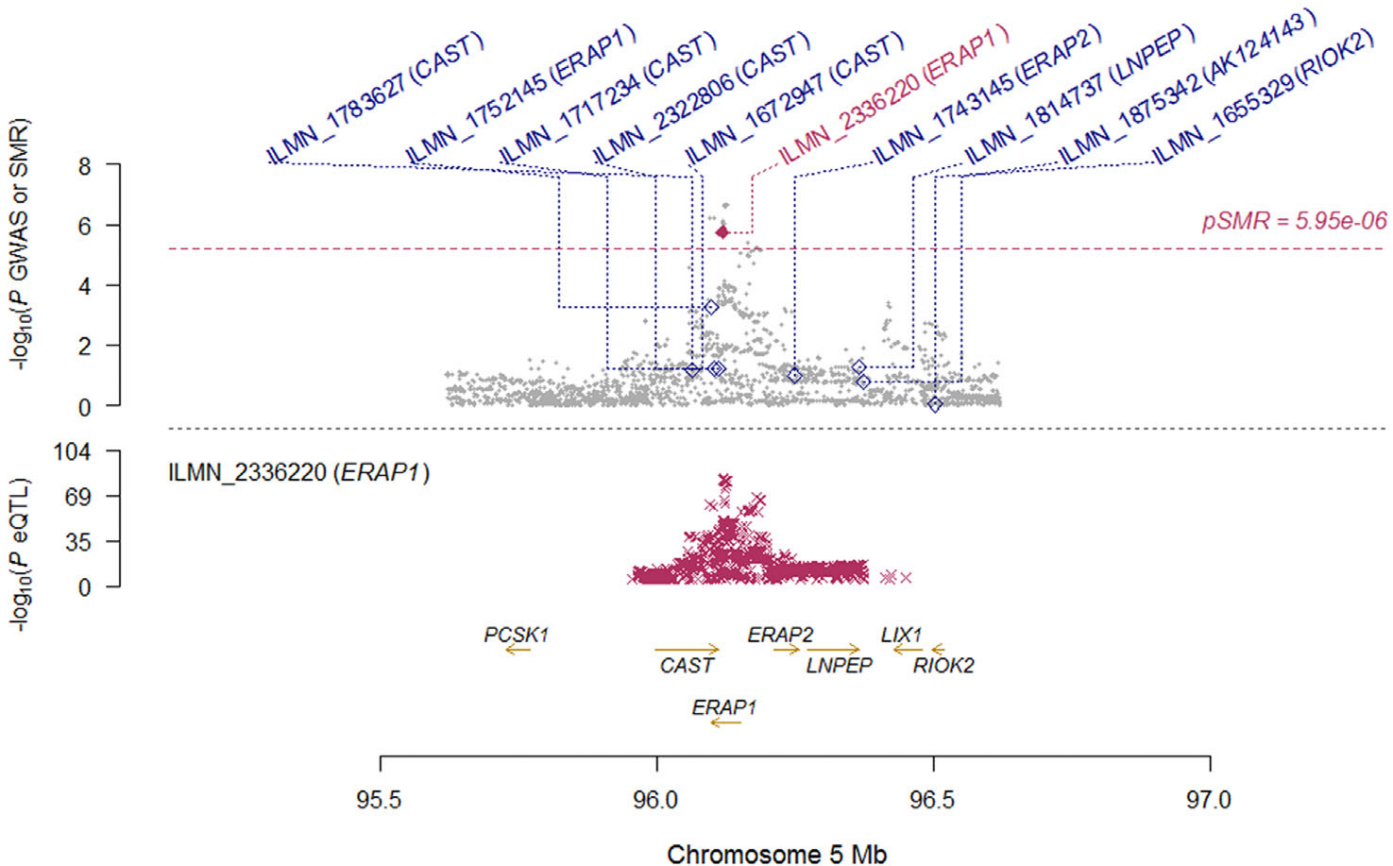
Locus zoom plot of SMR analysis for *ERAP1* locus. Top plot, grey dots represent the *P* values for SNPs from the comparison between AS+AAU+ and AS+AAU-, diamonds represent the *P* values for probes from the SMR test. Bottom plot, the eQTL *P* values of SNPs from the CAGE study for the ILMN_2336220 probe tagging *ERAP1*.

**Figure 4.**
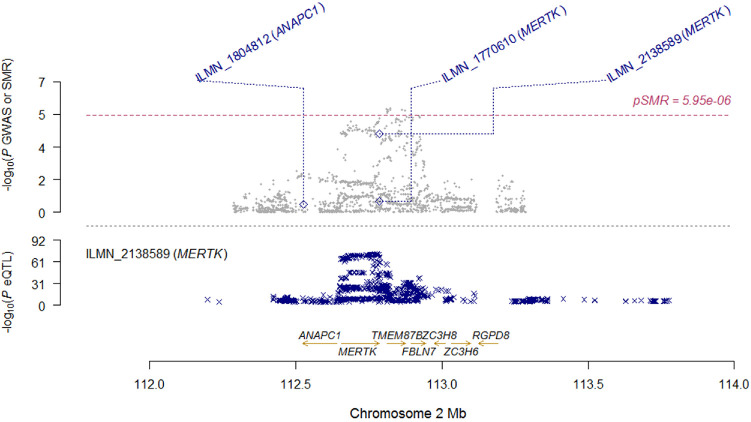
Locus zoom plot of SMR analysis for *MERTK* locus. Top plot, grey dots represent the *P* values for SNPs from the comparison between AS+AAU+ and AS+AAU-, diamonds represent the *P* values for probes from the SMR test. Bottom plot, the eQTL *P* values of SNPs from the CAGE study for the ILMN_2138589 probe tagging *MERTK*.

Results of top signals are summarized in Table 3. Notably, the most significant signal was *ERAP1* (*P*_GWAS_ = 7.91 × 10^−7^, *P*_eQTL_ = 4.71 × 10^−83^, *P*_SMR_ = 1.75 × 10^−6^), which reached genomewide significance (Table 3 and Fig. 3). In addition to *ERAP1*, the most associated gene was *MERTK* (*P*_GWAS_ = 3.95 × 10^−5^, *P*_eQTL_ = 4.02 × 10^−73^, *P*_SMR_ = 6.25 × 10^−5^) which was very close to genomewide significance (Table 3 and Fig. 4). These findings indicate that *ERAP1* and *MERTK* are the most functionally relevant genes in these two loci.

**Table 3.** Results of Summary Data-Based Mendelian Randomization Analysis for AAU with eQTL Data

							GWAS		eQTL		SMR	
Probe ID	Chr.	Nearby Genes	Probe Position^*^	Top SNP	SNP Position^*^	Effect Allele	Effect Size	*P* Value	Effect Size	*P* Value	Effect Size	*P* Value
ILMN_2336220	5	*ERAP1*	96118859	rs39841	96120170	G	0.193	7.91 × 10^−7^	0.593	4.71 × 10^−83^	0.325	1.75 × 10^−6^
ILMN_2138589	2	*MERTK*	112786554	rs78116208	112769801	T	0.169	3.95 × 10^−5^	−0.558	4.02 × 10^−73^	−0.304	6.25 × 10^−5^
ILMN_1723116	16	*AMFR*	56395803	rs11644357	56445252	G	−0.138	1.09 × 10^−4^	−0.920	2.71 × 10^−275^	0.150	1.19 × 10^−4^
ILMN_1664912	9	*IL11RA*	34661803	rs2070074	34649442	G	0.203	4.28 × 10^−4^	1.290	9.16 × 10^−144^	0.157	4.95 × 10^−4^
ILMN_1769550	17	*SLFN5*	33593671	rs12602385	33563756	T	0.129	4.02 × 10^−4^	0.464	4.90 × 10^−61^	0.278	5.29 × 10^−4^
ILMN_1752145	5	*ERAP1*	96098078	rs1057569	96109610	A	−0.150	4.29 × 10^−4^	0.562	3.88 × 10^−75^	−0.267	5.43 × 10^−4^
ILMN_1657475	9	*GALT*	34650419	rs2070074	34649442	G	0.203	4.28 × 10^−4^	−0.827	4.79 × 10^−63^	−0.246	5.79 × 10^−4^
ILMN_1774761	3	*CCR2*	46401979	rs2172247	46214670	T	0.131	3.06 × 10^−4^	−0.254	4.88 × 10^−20^	−0.518	8.03 × 10^−4^
ILMN_1720024	9	*IL11RA*	34660552	rs2070074	34649442	G	0.203	4.28 × 10^−4^	0.522	4.85 × 10^−26^	0.389	8.50 × 10^−4^

Chr., chromosome; GWAS, genomewide association study; eQTL, expression quantitative trait loci; SMR, summary data-based Mendelian randomization.

* UCSC, human genome build 19.

### Estimation of SNP-Based Heritability

Heritability is the proportion of the phenotypic variance accounted for by genetic effects. We investigated SNP-based heritability by designing three different comparisons: (1) patients with AS with AAU versus AS alone; (2) patients with AS alone versus controls; and (3) patients with AS with AAU versus controls. The details are summarized in Table 4. The estimation of heritability was 0.40 to 0.43 in the comparison between patients with AS with AAU and patients with AS without AAU, adjusting prevalence from 0.3 to 0.5. Interestingly, in the comparison between AS alone and controls, the estimated heritability was 0.48 to 0.60, adjusting prevalence from 0.002 to 0.006. When we compared the patients with AS and with AAU to controls, the estimated heritability was 0.59 to 0.72, adjusting prevalence from 0.001 to 0.003. These discoveries not only confirmed that AS and AAU are highly heritably disorders, but also suggest that there are additional genetic contributors to AAU compared with AS alone.

**Table 4.** Estimation of Heritability in Three Comparisons

Cases (Number)	Controls (Number)	Heritability* (SE; Adjusted Prevalence)		
AS+AAU+ (2752)	AS+AAU− (3836)	0.40 (0.071; 0.3)	0.42 (0.075; 0.4)	0.43 (0.077; 0.5)
AS+AAU− (3836)	Controls (14542)	0.48 (0.008; 0.002)	0.55 (0.010; 0.004)	0.60 (0.010; 0.006)
AS+AAU+ (2752)	Controls (14542)	0.59 (0.009; 0.001)	0.67 (0.010; 0.002)	0.72 (0.011; 0.003)

* Heritability is calculated using GCTA (https://cnsgenomics.com/software/gcta/#Download).

### Genetic Risk Prediction

The greater heritability of AS+AAU+ compared with AS alone or AS+AAU- suggests that PRS have the potential to identify AAU cases likely to develop AS, and conversely AS cases likely to develop AAU. To assess the discriminatory capacity and accuracy in risk prediction, we first performed the analyses of PRS by using two different designs (1) AS+AAU+ versus AS+AAU-; and (2) AS+AAU+ versus controls. In the first comparison, AS+AAU+ versus AS+AAU-, the overall discriminatory capacity of PRS was weak (AUC = 0.56; 95% CI = 0.54–0.58; *P* value = 2.10 × 10^−10^). Assuming the prevalence of patients with AS with AAU is 40% among the AS population, those patients with AS in the top 50% of genetic risk had an estimated genetic risk of developing AAU of 43.3% (SD = 0.9%; Supplementary Fig. S3). Those in the bottom 50% had < 37% chance of developing the disease. Thus, in the setting of a patient with AS, PRS alone are currently not helpful in distinguishing those patients likely to develop AAU.

**Figure 5.**
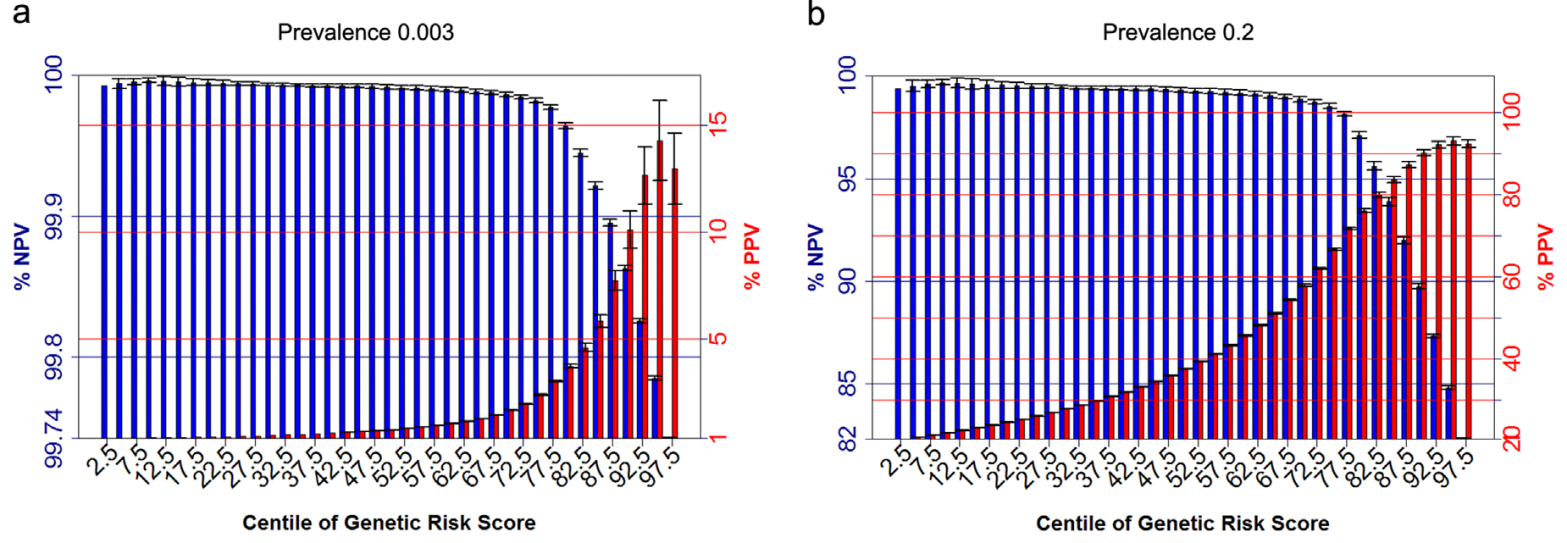
Positive and negative predictive values for patients with AS with AAU for centiles of genetic risk scores. The assumed prevalence of patients with AS with AAU of 0.3% among the population (**a**), and assumed prevalence of 20% in outpatient clinics (**b**). Error Bars denote 2 standard deviations based on 10-fold cross validation.

In the second comparison, AS+AAU+ versus controls, we observed the overall discriminatory capacity of genomewide PRS was very strong (AUC = 0.96; 95% CI = 0.955–0.966). When computing PRS using HLA-B27 alone, the AUC is 0.92 (95% CI = 0.915–0.927). Considering a general population setting, assuming the prevalence of patients with AS with AAU is 0.3% among the population, those in the top 10% of genetic risk had an estimated genetic risk of developing AS with AAU of 10.1% (SD = 0.9%; Fig. 5a), 2.9 times higher than the risk using HLA-B27 alone (3.5% [SD = 0.1%], Supplementary Fig. S4a). Those in the top 5% had a risk of developing AS with AAU of 14.3% (SD = 1.9%; Fig. 5a), 3.6 × higher than the risk estimate using HLA-B27 alone (4% [SD = 0.4%]). Those in the bottom 85% had < 0.1% chance of developing the disease, similar to the estimate using HLA-B27 alone. Considering a clinical setting where patients with AAU are being assessed for their risk of AS, assuming the prevalence of patients with AS with AAU is 20% in outpatient clinics, we observed the estimated genetic risk of developing disease of 90.3% (SD = 0.8%) for those in the top 10% and 93.2% (SD = 1.0%) for those in the top 5% (Fig. 5b). Using HLA-B27 alone, the estimated genetic risk of developing disease of 75.2% (SD = 0.9%) for those in the top 10% and 77.3% (SD = 1.9%). Considering negative predictive values, using the PRS the bottom 65% of patients would have < 1% chance of also having AS. The performance using a score for HLA-B27 alone is marginally worse, with the maximum negative predictive value (NPV) being 98%, 75% of the distribution (Supplementary Fig. S4b). These results indicate that genomewide PRS has better performance than HLA-B27 alone, particularly in positive predictive value (PPV) analyses.

## Discussion

This study has expanded understanding of the genetics of AAU, through the discovery of novel suggestive associated loci including *MERTK*, *CLCN7*, *ACAA2*, *KIFAP3*, and five intergenic loci. Among those new loci, *MERTK* locus is also a novel finding in our ongoing GWAS in AS. Interestingly, Mendelian randomization analysis for AAU with eQTL data showed that *MERTK* is the most functionally relevant gene in the locus.

MERTK is a member of the TYRO3/AXL/MER (TAM) receptor kinase family and encodes a transmembrane protein, which has fibronectin type-III domains, two immunoglobulin-like (Ig-like) C2-type domains, and one tyrosine kinase domain.^41^ The three TAM receptors interact functionally with one another in their pleiotropic roles in immune regulation, including in autoimmune and infectious diseases, and cancer.^42^ *MERTK* was not previously known to be associated with AS or AAU, whereas mutations in *MERTK* gene lead to an inherited retinal disorder, named retinitis pigmentosa.^41^ Notably, mutations in previously reported AAU-related gene *EYS* also lead to retinitis pigmentosa.^9^^,^^43^ In addition to retinitis pigmentosa, genetic associations of *MERTK* have also been reported in a few other traits. SNP associations with SNPs in linkage disequilibrium with the strongest associated SNP in AAU have previously been reported in multiple sclerosis and systolic blood pressure (r2 > 0.5, both concordant in direction), and with other SNPs not in linkage disequilibrium (r2 < 0.3) with rs10171979, including hepatitis C induced liver fibrosis, coronary artery disease, heel bone mineral density, and white blood cell count (https://www.ebi.ac.uk/gwas/genes/MERTK). Axl/Mertk double knockout mice are susceptible to T-cell mediated uveitis,^44^ and Tyro3/Axl/Mertk triple knockout mice develop bone marrow edema and macrophage and B-cell and T-cell infiltration, consistent with changes seen in spondyloarthritidies.^45^ MERTK is expressed at high levels in the ovaries, prostate, testis, lungs, retinas, and kidneys, and at lower levels in the heart, brain, and skeletal muscle.^46^ MERTK is also expressed in macrophages, dendritic cells, natural killer (NK) cells, NKT cells, and platelets.^46^^–^^48^ MERTK is not expressed on the normal lymphocytes but has been found to be expressed in a majority of lymphoblasts from patients with T-cell leukemia, certain subsets of B-cell leukemia, and mantle cell lymphoma.^49^^,^^50^ Additionally, MERTK signaling plays a role in various processes, such as macrophage clearance of apoptotic cells, platelet aggregation, cytoskeleton reorganization, and engulfment. Animal models that lack functional MERTK protein have macrophages that are unable to appropriately engulf apoptotic cells. This inefficient clearance of dead cells can lead to activation of inflammation and development of autoimmunity.^51^ Moreover, MERTK was also reported as a potent suppressor of T cell response.^52^ Taken together, these discoveries indicate the potential role of *MERTK* in the etiology of AAU. However, the functional relevance in this study was based on gene expression and did not include other functions. Future functional studies are necessary to further understand the biological mechanisms underpinning the association of *MERTK* with AAU.

This study also confirmed multiple discoveries from previous genetics of AS/AAU. We confirmed the association of previously reported loci associated with AAU from a large genetic study on the basis of immunochip genotyping, including *HLA-B*, *ERAP1*, *IL23R*, and *IL10*.^9^ We also confirmed the association of previously reported genetic loci associated with AS but not yet with AAU, including *NOS2*, *ASAP2*, *CMC1*, *IL12B*, *ZC3H12C*, *SP140*, and *PTPN2*. Furthermore, consistent with previous studies on MHC loci of AS/AAU, HLA imputation of this study showed that HLA-B*27 was the most significantly associated allele with AAU (Supplementary Fig. S5a), and we also observed HLA-B*27 homozygosity increases risk over heterozygosity. We also confirmed the existence of an interaction between *ERAP1* and HLA-B*27 for AAU, which was initially discovered in a GWAS of AS. Minor additional risk association was seen with HLA-DRB1*15 (Supplementary Fig. S5b), consistent with previous reports using direct genotyping,^53^ and with HLA-DPB1*03 (Supplementary Fig. S5c), independent of *HLA-B27*, suggesting the presence of additional MHC encoded factors influencing AAU risk in patients with AS.

We have estimated SNP-based heritability in different comparisons, and found both patients with AS alone and patients with AS and with AAU showed high heritability. The estimations of heritability based on genotyped SNPs reached approximately 0.7, which is very close to the result from twin studies for AS alone (heritability > 0.9).^54^^,^^55^ Moreover, the AS+AAU+ cohort showed higher heritability than the AS+AAU- cohort, indicating that AAU cases carry additional genetic risk compared with cases with isolated AS. This supports further initiatives to identify AAU genetic susceptibility factors.

To assess the discriminatory capacity and accuracy in risk prediction using genomewide PRS, we first applied PRS on subjects with AS+AAU+ versus subjects with AS alone. But the overall discriminatory capacity of this PRS (AUC = 0.56) was too low to be of clinical utility. Subsequently, we applied PRS on subjects with AS+AAU+ versus controls. Interestingly, we observed the discriminatory capacity of PRS was very strong with AUC of 0.96, and higher than the discriminatory capacity of HLA-B27 alone (AUC = 0.92). The difference in performance of the PRS and HLA-B27 alone is more apparent when PPV and NPVs are considered. As HLA-B27 is only carried in approximately 8% of European-descent populations, it can only demonstrate increased risk in that small group, and provides no information about the relative risk of the disease across the remaining 92% of the population. In contrast, not only does the PRS provide higher PPVs even among HLA-B27 positive individuals, it also is informative about differences in AAU risk across HLA-B27 negative individuals. PRS also performed significantly better in excluding AS among patients with AAU than did HLA-B27 alone. These results suggest that PRS has significant potential clinical benefit in predicting the likelihood of developing AS with AAU, although it had limited capacity in risk prediction of the patients with AS developing AAU.

The prevalence of AAU increases almost linearly with increasing disease duration, with over 50% of patients with AS with > 40 years of disease duration having experienced AAU.^9^ Thus, it is likely that many patients classified in the current study as not being AAU affected will ultimately likely develop the complication. This reduces the power to detect genetic associations, to identify heritability, and to develop PRS. A further complication is the need to use study designs controlling for fact that these patients have an additional highly heritable disease, AS. The study design, therefore, is only able to distinguish factors that influence the risk of AAU over and above their effect on the risk of AS. Thus genetic effects on risk of AAU that are shared at similar strengths with AS will not be detected by this study design. Such associations could potentially be identified in studies comparing AAU cases not affected by AS with controls. However, the high prevalence of AS among AAU cases, and the high frequency of subclinical sacroiliitis in these patients, would mean that careful screening of cases would be required, and that cases would need to be old enough that AS would be unlikely to develop in the future if it had not already manifested. We also acknowledge the limitation of our study of lack of independent replication to test the observed associations across multiple cohorts. Future studies should consider our GWAS findings in different populations.

In conclusion, we report here the first GWAS for AAU and identified new susceptibility loci. The findings of association with the *HLA-B*, *ERAP1*, *NOS2*, and *IL23R* loci are consistent with a strong overlap in etiopathogenesis with AS. We identified new suggestive associated loci of AAU, including *MERTK*, which was reported to be involved in the development of autoimmunity, indicating the potential role of *MERTK* in the etiology of AAU. Further research is needed into the immunopathogenic mechanisms of AAU and AS. Investigation of genomewide polygenic risk scores of AS alone and AS with AAU both showed strong discriminatory capacity and high accuracy in risk prediction. Our findings suggest PRS based on GWAS data can quantify individual AS and AAU risks in clinically significant ways, potentially leading to the effective implementation of genetic discoveries in healthcare applications.

## Supplementary Material

Supplement 1

Supplement 2

Supplement 3

Supplement 4

Supplement 5

Supplement 6
