# Supplementary material for: Genomewide Association Study of Acute Anterior Uveitis Identifies New Susceptibility Loci
Source: Invest Ophthalmol Vis Sci. 2020 Jun 3;61(6):3. doi: 10.1167/iovs.61.6.3 (PMC7415282; doi:10.1167/iovs.61.6.3)
Supplement: Supplement 1 [file iovs-61-6-3_s001.pdf]

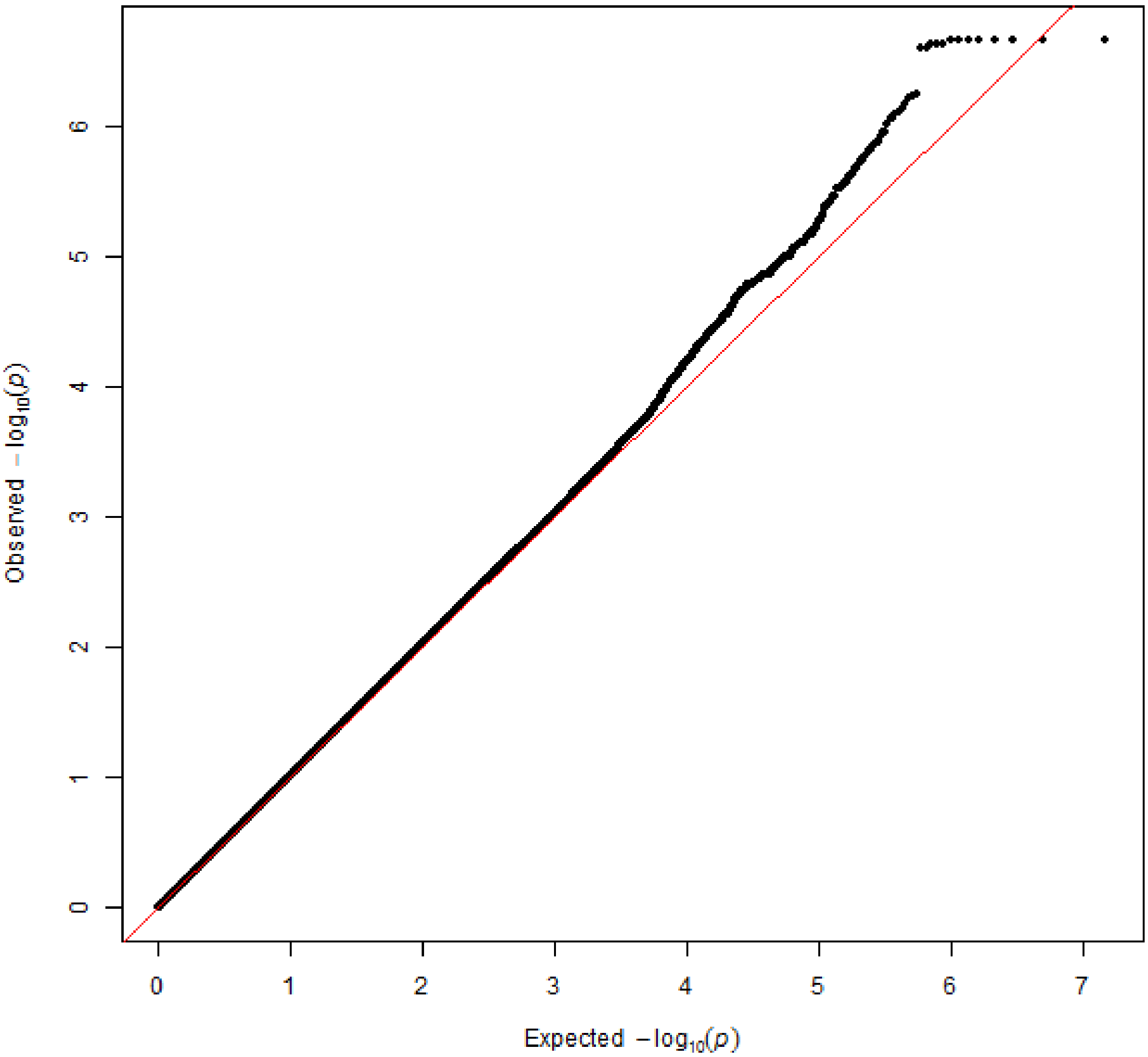

Figure S1. Quantile-quantile plot of association for the comparison of AS patients with AAU versus AS patients without AAU. MHC SNPs were excluded.
