# Supplementary figures and images for: Genomewide Association Study of Acute Anterior Uveitis Identifies New Susceptibility Loci
Source: Invest Ophthalmol Vis Sci. 2020 Jun 3;61(6):3. doi: 10.1167/iovs.61.6.3 (PMC7415282; doi:10.1167/iovs.61.6.3)

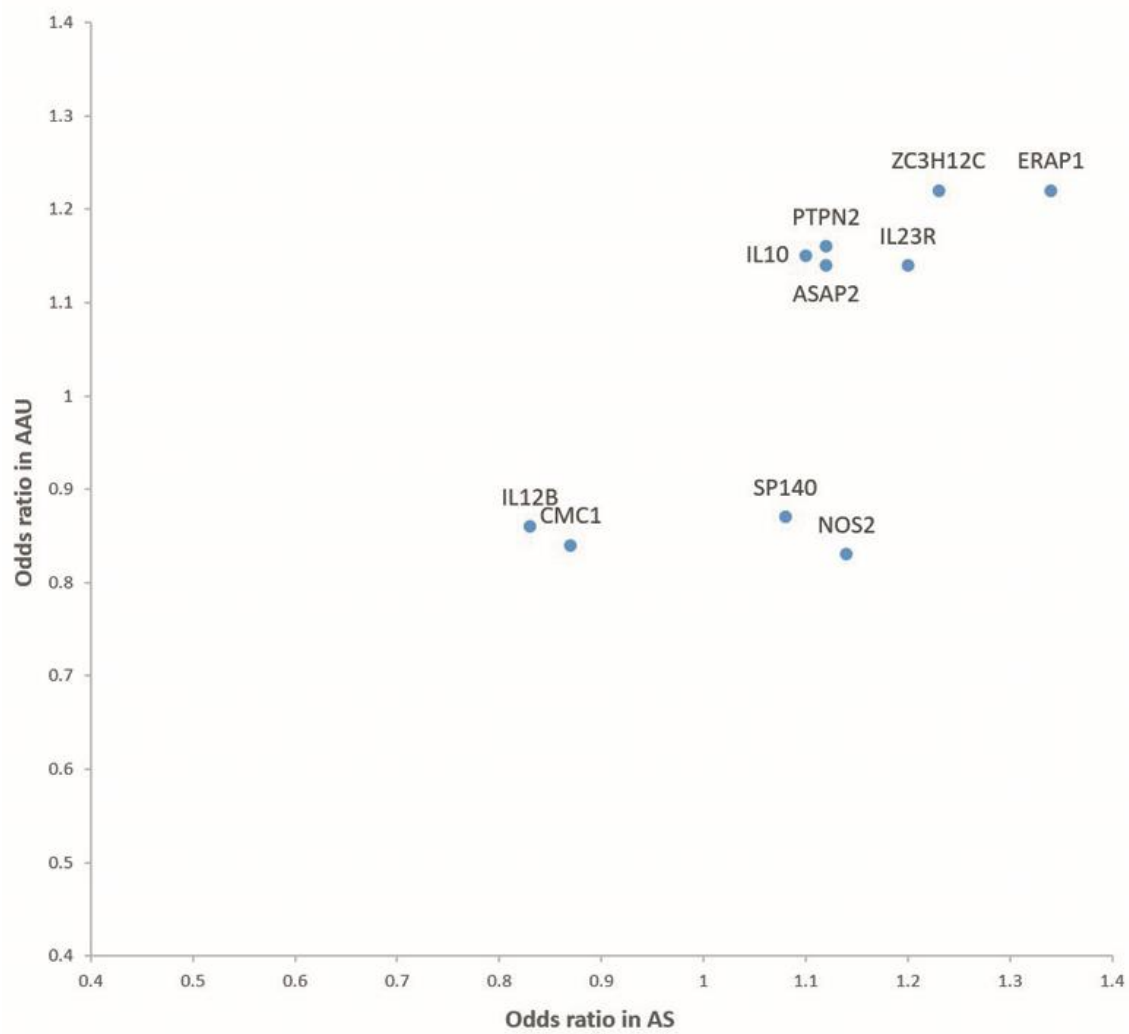

**Figure S2.** Comparison of odd ration of reported AS genes in AAU and AS.

Supplement: Supplement 2 [file iovs-61-6-3_s002.pdf]
