# Supplementary material for: Genomewide Association Study of Acute Anterior Uveitis Identifies New Susceptibility Loci
Source: Invest Ophthalmol Vis Sci. 2020 Jun 3;61(6):3. doi: 10.1167/iovs.61.6.3 (PMC7415282; doi:10.1167/iovs.61.6.3)
Supplement: Supplement 3 [file iovs-61-6-3_s003.pdf]

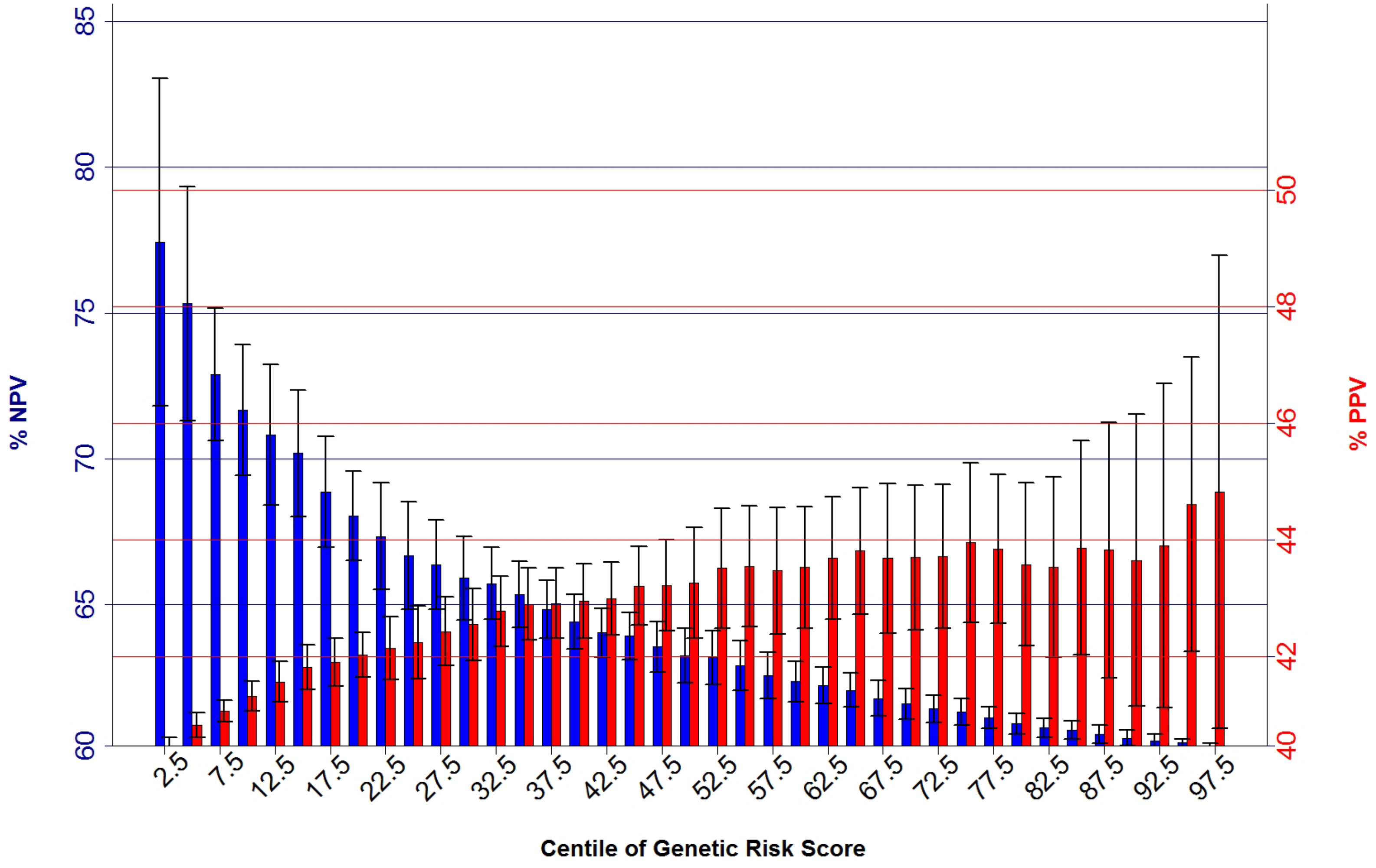

Figure S3. Positive and negative predictive values for AS+AAU+ versus AS+AAU- for centiles of genetic risk scores. The assumed prevalence of AS patients with AAU of 40% amongst the AS population. Error Bars denote 2 standard deviations based on 10-fold cross validation.
