# Supplementary material for: Genomewide Association Study of Acute Anterior Uveitis Identifies New Susceptibility Loci
Source: Invest Ophthalmol Vis Sci. 2020 Jun 3;61(6):3. doi: 10.1167/iovs.61.6.3 (PMC7415282; doi:10.1167/iovs.61.6.3)
Supplement: Supplement 4 [file iovs-61-6-3_s004.pdf]

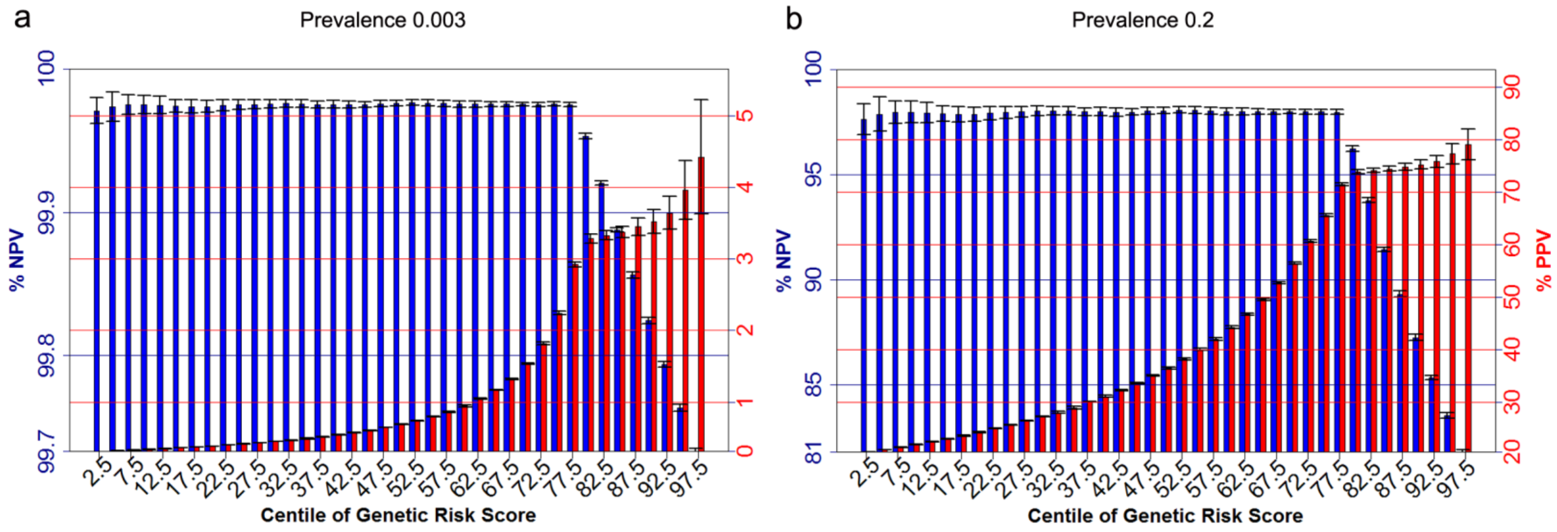

Figure S4. Positive and negative predictive values of B27 alone for AS patients with AAU for centiles of genetic risk scores. The assumed prevalence of AS patients with AAU of 0.3% amongst the population (a), and assumed prevalence of 20% in outpatient clinics (b). Error Bars denote 2 standard deviations based on 10-fold cross validation.
