# Supplementary material for: Genomewide Association Study of Acute Anterior Uveitis Identifies New Susceptibility Loci
Source: Invest Ophthalmol Vis Sci. 2020 Jun 3;61(6):3. doi: 10.1167/iovs.61.6.3 (PMC7415282; doi:10.1167/iovs.61.6.3)
Supplement: Supplement 5 [file iovs-61-6-3_s005.pdf]

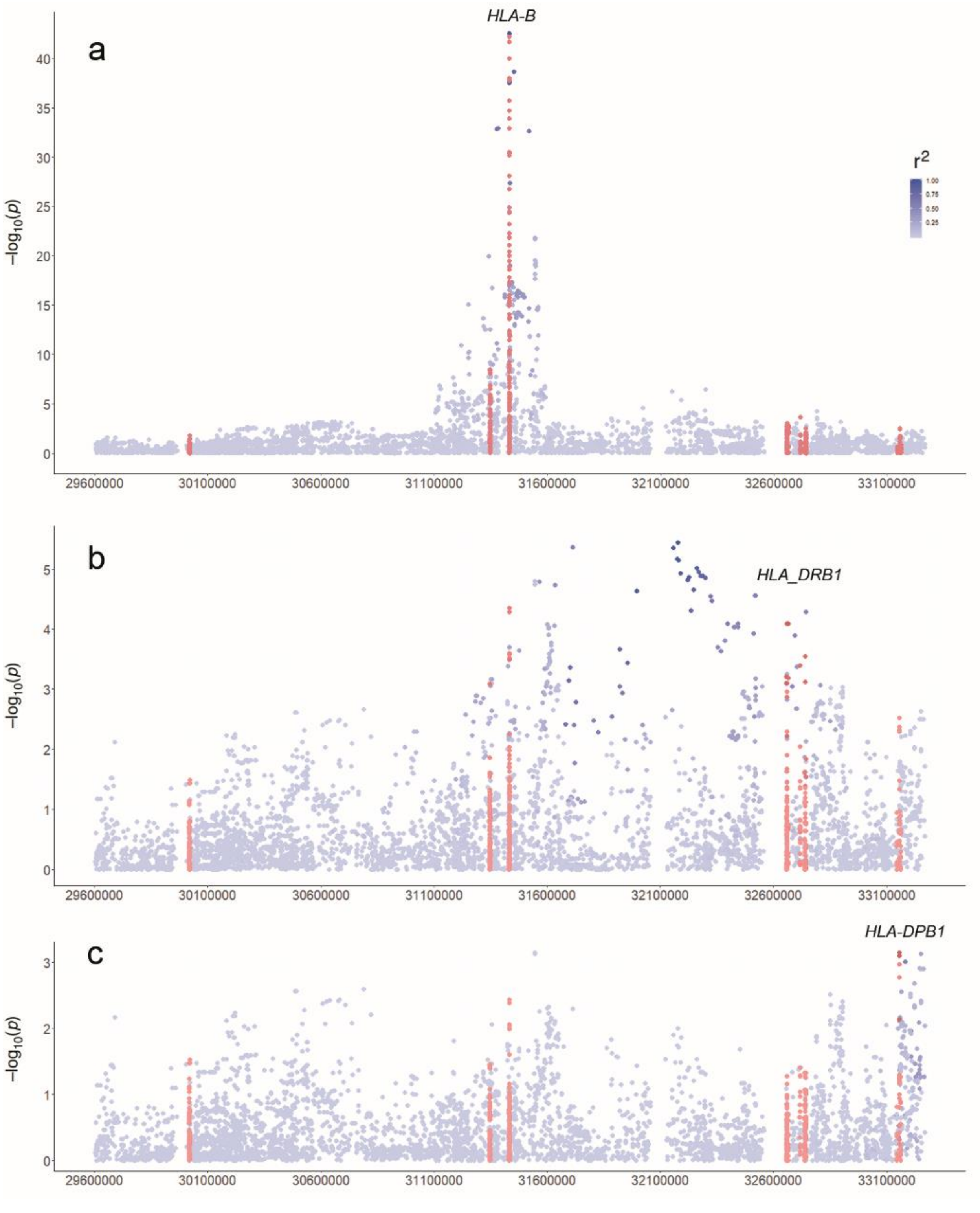

Figure S5. Manhattan plot of the conditional testing across the MHC. (a) The strongest association was found with SNPs and amino-acid position in the *HLA-B* locus. (b) Controlling for the effect of *HLA-B*

susceptibility alleles, an independent association was observed with SNPs and amino-acid position in the *HLA-DRB1* locus. (c) Controlling for the effect of both *HLA-B* and *HLA-DRB1* susceptibility alleles, an independent association was observed with SNPs and amino-acid position in the *HLA-DPB1* locus. SNP association tests are shown in blue circles, colour coded by linkage disequilibrium from the SNP with the strongest association. Amino-acid position tests are shown as red circles.
