# Supplementary material for: Genomewide Association Study of Acute Anterior Uveitis Identifies New Susceptibility Loci
Source: Invest Ophthalmol Vis Sci. 2020 Jun 3;61(6):3. doi: 10.1167/iovs.61.6.3 (PMC7415282; doi:10.1167/iovs.61.6.3)
Supplement: Supplement 6 [file iovs-61-6-3_s006.pdf]

Table S1 AS cases and healthy controls involved in this study.

Type	Cohort	Number
AS	United Kingdom	5,522
AS	Australo-Anglo-American Spondyloarthritis Consortium (TASC)	703
AS	Groupe Française d'Etude Génétique des Spondylarthrites (GFEGS)	191
AS	Australian	172
Total		*6,588*
Controls	Understanding Society – The UK Household Longitudinal Study[1]	9,469
Controls	Queensland Institute of Medical Research Berghofer Medical Research Institute Twins Cohort[2]	2,664
Controls	Advancing Exercise & Sports Science Collaborative Research Network[3]	1,706
Controls	Australian general population[4]	621
Controls	Oregon Metagenomics Controls[5]	82
Total		*14,542*

1. https://www.understandingsociety.ac.uk/
2. https://www.qimrberghofer.edu.au/qtwin/
3. Australian recreational joggers not screened for any medical condition.
4. ‘Aussie Normals’ cohort (Koerbin et al, *Pathology*. 49(5):518-525, 2017.)
5. Asquith et al, *Arthritis Rheumatol*. Epub Apr 3, 2019.

Table S2 Summary of quality control.

**Thresholds**	
Genotyping missingness rate	0.05
Individual missingness rate	0.05
Hardy-Weinberg Equilibrium	1×10^{-6}
Minor allele frequency	0.01
Heterozygosity versus missingness outliers beyond 3 standard deviations (SD) were excluded.	
Identity by descent	0.185
**After QC**	
Individuals	6588
SNPs	7,436,415

Table S3 Genomic inflation factor stratified by frequency, imputation quality score and MHC.

**Region**	**λ**	**λ_{1000}**	**SNP set**
With MHC	1.0108	1.0034	Info 0.6 + MAF >1%
Non-MHC	1.0079	1.0024	Info 0.6 + MAF >1%
With MHC	1.0150	1.0047	Info 0.6 + MAF >5%
Non-MHC	1.0131	1.0041	Info 0.6 + MAF >5%
With MHC	1.0080	1.0024	Info 0.8 + MAF >1%
Non-MHC	1.0065	1.0020	Info 0.8 + MAF >1%
With MHC	1.0150	1.0047	Info 0.8 + MAF >5%
Non-MHC	1.0131	1.0041	Info 0.8 + MAF >5%

λ, genomic inflation factor; Info, imputation quality score; MAF, minor allele frequency.

Table S4 Results of the associations between the causal genes of AS and AAU.

Reported AS genes	SNP ID	Chr.	Position*	*P*	Effect allele	OR	95% CI	Tag SNP in AS# (OR_{AS}/OR_{AAU})	D'/R^2	Direction in AS
IL23R	rs11209032	1	67740092	2.55×10^{-4}	A	1.14	1.06-1.23	rs11209032 (1.20/1.14)	1/1	Concordant
ASAP2	rs56111045	2	9406008	7.98×10^{-4}	G	1.14	1.06-1.23	rs2666218 (1.12/1.13)	1/0.995	Concordant
CMC1	rs10510609	3	28305301	4.10×10^{-4}	A	0.84	0.76-0.93	rs10510607 (0.87/0.85)	0.965/0.913	Concordant
IL12B	rs2853696	5	158744660	8.91×10^{-4}	T	0.86	0.78-0.94	rs6556416 (0.83/0.89)	0.778/0.277	Concordant
ZC3H12C	rs17645205	11	109981505	6.34×10^{-4}	G	1.22	1.09-1.37	rs7115956 (1.23/1.001)	0.953/0.086	Concordant
IL10	rs6703630	1	206948639	4.07×10^{-4}	T	1.15	1.06-1.24	rs3024493 (1.1/0.997)	0.846/0.043	ND
SP140	rs10498245	2	231111422	4.07×10^{-4}	G	0.87	0.81-0.94	rs12694846 (1.08/0.927)	0.556/0.231	ND
PTPN2	rs8084448	18	12888220	2.91×10^{-4}	T	1.16	1.07-1.26	rs12968719 (1.12/1.11)	0.307/0.03	ND

Chr., chromosome; OR, odds ratio; 95% CI, 95% confidence interval; ND, not determined.

* UCSC human genome build 19.

The list of loci associated with AS was from the recent published review of AS genetics.[24]

Table S5 Association signals and amino acid positions in HLA proteins.

Chr.	Amino acid position	OR	L95	U95	*P*-value
6	AA_B_97_31432180_N	2.35	2.08	2.66	6.09×10^{-43}
6	AA_B_70_31432506_K	2.35	2.07	2.65	2.20×10^{-42}
6	AA_B_114_31432129_DN	2.27	2.01	2.56	9.84×10^{-41}
6	AA_B_114_31432129_H	2.27	2.01	2.56	9.84×10^{-41}
6	AA_B_114_31432129_HK	2.27	2.01	2.56	9.84×10^{-41}
6	AA_B_114_31432129_Hx	2.27	2.01	2.56	9.84×10^{-41}
6	AA_B_77_31432485_D	2.16	1.92	2.42	1.10×10^{-38}
6	AA_B_77_31432485_SN	2.15	1.92	2.42	1.60×10^{-38}
6	AA_B_97_31432180_NV	2.09	1.86	2.35	1.96×10^{-36}
6	AA_B_70_31432506_QK	0.54	0.49	0.60	1.89×10^{-35}
6	AA_B_70_31432506_QN	2.03	1.82	2.28	1.18×10^{-34}
6	AA_B_97_31432180_WN	1.96	1.76	2.19	1.16×10^{-33}
6	AA_B_69_31432509_A	0.57	0.52	0.63	3.29×10^{-31}
6	AA_B_69_31432509_T	0.58	0.52	0.63	6.33×10^{-31}
6	AA_B_70_31432506_N	0.58	0.52	0.63	6.33×10^{-31}
6	AA_B_71_31432503	0.58	0.52	0.63	6.33×10^{-31}
6	AA_B_97_31432180_SRT	1.81	1.63	2.01	8.04×10^{-29}
6	AA_B_97_31432180_TN	1.73	1.57	1.92	1.65×10^{-27}
6	AA_B_67_31432515_C	1.73	1.56	1.91	1.35×10^{-25}
6	AA_B_67_31432515_YC	0.62	0.57	0.68	4.25×10^{-25}
6	AA_B_97_31432180_SRW	0.60	0.55	0.66	5.96×10^{-24}
6	AA_B_163_31431982_E	0.64	0.58	0.70	5.46×10^{-23}
6	AA_B_163_31431982_Ex	0.64	0.58	0.70	5.46×10^{-23}
6	AA_B_11_31432683	0.63	0.58	0.69	1.34×10^{-22}
6	AA_B_97_31432180_SRV	0.62	0.56	0.68	1.56×10^{-22}
6	AA_B_67_31432515_FS	0.65	0.59	0.71	7.71×10^{-22}
6	AA_B_67_31432515_CM	1.60	1.45	1.77	3.69×10^{-21}
6	AA_B_97_31432180_SN	0.66	0.60	0.72	1.01×10^{-20}
6	AA_B_12_31432680	0.65	0.60	0.72	3.19×10^{-20}
6	AA_B_97_31432180_SR	0.65	0.59	0.71	1.24×10^{-19}
6	AA_B_116_31432123_D	0.65	0.60	0.72	2.30×10^{-19}
6	AA_B_116_31432123_Dx	0.65	0.60	0.72	2.30×10^{-19}
6	AA_B_97_31432180_SNV	0.67	0.62	0.73	1.45×10^{-18}

6	AA_B_9_31432689_H	0.67	0.61	0.73	4.90×10^{-18}
6	AA_B_97_31432180_SWN	0.68	0.63	0.74	6.65×10^{-18}
6	AA_B_74_31432494	0.70	0.64	0.76	9.47×10^{-17}
6	AA_B_97_31432180_STN	0.69	0.64	0.76	1.76×10^{-16}
6	AA_B_80_31432476_T	0.69	0.63	0.75	3.19×10^{-16}
6	AA_B_116_31432123_DL	0.69	0.63	0.75	3.94×10^{-16}
6	AA_B_116_31432123_YFS	0.69	0.63	0.75	3.94×10^{-16}
6	AA_B_97_31432180_RT	0.70	0.64	0.76	5.42×10^{-16}
6	AA_B_45_31432581_E	0.71	0.65	0.77	1.25×10^{-15}
6	AA_B_45_31432581_EG	0.71	0.65	0.77	1.25×10^{-15}
6	AA_B_97_31432180_RW	0.71	0.65	0.77	8.30×10^{-15}
6	AA_B_113_31432132_H	0.71	0.65	0.77	1.94×10^{-14}
6	AA_B_113_31432132_Y	0.71	0.65	0.77	1.94×10^{-14}
6	AA_B_97_31432180_RV	0.71	0.65	0.78	2.50×10^{-14}
6	AA_B_116_31432123_FD	0.72	0.66	0.79	3.94×10^{-13}
6	AA_B_116_31432123_YLS	0.72	0.66	0.79	3.94×10^{-13}
6	AA_B_97_31432180_R	0.73	0.67	0.79	8.19×10^{-13}
6	AA_B_-16_31432889_L	0.73	0.66	0.79	1.32×10^{-12}
6	AA_B_-16_31432889_V	0.73	0.66	0.79	1.32×10^{-12}
6	AA_B_32_31432620	0.73	0.67	0.80	3.43×10^{-12}
6	AA_B_116_31432123_YS	0.75	0.69	0.81	3.81×10^{-11}
6	AA_B_116_31432123_YSx	0.75	0.69	0.81	3.81×10^{-11}
6	AA_B_77_31432485_SG	0.74	0.68	0.81	6.50×10^{-11}
6	AA_B_67_31432515_SM	0.75	0.69	0.81	6.77×10^{-11}
6	AA_B_77_31432485_S	0.74	0.68	0.81	7.44×10^{-11}
6	AA_B_80_31432476_N	0.75	0.68	0.81	8.10×10^{-11}
6	AA_B_82_31432470	0.75	0.68	0.81	8.10×10^{-11}
6	AA_B_83_31432467	0.75	0.68	0.81	8.10×10^{-11}
6	AA_B_24_31432644_T	0.75	0.69	0.82	9.62×10^{-11}
6	AA_B_9_31432689_Y	0.76	0.70	0.83	5.23×10^{-10}
6	AA_B_45_31432581_EM	0.76	0.70	0.83	1.26×10^{-9}
6	AA_B_45_31432581_TK	0.76	0.70	0.83	1.26×10^{-9}
6	AA_B_67_31432515_S	0.76	0.70	0.83	2.31×10^{-9}
6	AA_C_16_31347579	1.32	1.20	1.45	3.37×10^{-9}
6	AA_C_211_31346157_T	1.32	1.20	1.45	3.37×10^{-9}

6	AA_B_114_31432129_DH	0.77	0.70	0.84	4.13×10^{-9}
6	AA_B_114_31432129_N	0.77	0.70	0.84	4.13×10^{-9}
6	AA_B_114_31432129_NK	0.77	0.70	0.84	4.13×10^{-9}
6	AA_B_114_31432129_Nx	0.77	0.70	0.84	4.13×10^{-9}
6	AA_B_45_31432581_ET	0.76	0.70	0.83	4.66×10^{-9}
6	AA_B_45_31432581_KM	0.76	0.70	0.83	4.66×10^{-9}
6	AA_B_163_31431982_ET	0.76	0.70	0.84	4.92×10^{-9}
6	AA_B_163_31431982_L	0.76	0.70	0.84	4.92×10^{-9}
6	AA_B_116_31432123_YD	0.76	0.69	0.83	5.30×10^{-9}
6	AA_B_116_31432123_YDx	0.76	0.69	0.83	5.30×10^{-9}
6	AA_C_163_31346888_E	1.31	1.19	1.44	6.26×10^{-9}
6	AA_C_211_31346157_A	1.31	1.19	1.44	9.10×10^{-9}
6	AA_B_116_31432123_FS	0.75	0.68	0.83	1.82×10^{-8}
6	AA_B_116_31432123_YDL	0.75	0.68	0.83	1.82×10^{-8}
6	AA_B_156_31432003_DW	0.76	0.69	0.84	2.37×10^{-8}
6	AA_B_156_31432003_RL	0.76	0.69	0.84	2.37×10^{-8}
6	AA_B_325_31430282_C	0.77	0.70	0.84	3.20×10^{-8}
6	AA_B_325_31430282_S	0.77	0.70	0.84	4.28×10^{-8}

Chr., chromosome; OR, odds ratio; L95, Lower bound of 95% confidence interval for odds ratio; U95, Upper bound of 95% confidence interval for odds ratio.

Table S6 Association signals and amino acid positions in HLA proteins after adjusting for the top signal.

Chr.	Amino acid	OR	L95	U95	*P*
6	AA_B_67_31432515_Y	1.31	1.15	1.49	3.80×10^{-5}
6	AA_B_70_31432506_Q	1.30	1.15	1.49	4.42×10^{-5}
6	AA_B_70_31432506_QK	0.76	0.67	0.87	4.80×10^{-5}
6	AA_DRB1_71_32659935_A	1.27	1.13	1.44	8.51×10^{-5}
6	AA_DRB1_-1_32665412_S	1.27	1.13	1.44	8.51×10^{-5}

Chr., chromosome; OR, odds ratio; L95, Lower bound of 95% confidence interval for odds ratio; U95, Upper bound of 95% confidence interval for odds ratio.
